# Do we need orthogeriatrics in Poland? Changes in the age structure and location of hip fractures

**DOI:** 10.1007/s40520-016-0627-1

**Published:** 2016-09-06

**Authors:** Robert Wilk, Michał Skrzypek, Małgorzata Kowalska, Damian Kusz, Bogdan Koczy, Piotr Zagórski, Wojciech Pluskiewicz

**Affiliations:** 1 Department of Orthopedics and Traumatology, School of Medicine in Katowice, Medical University of Silesia in Katowice, Ziołowa 45/47 Street, 40-635 Katowice, Poland; 2 Department of Biostatistics, School of Public Health in Bytom, Medical University of Silesia in Katowice, Bytom, Poland; 3 Department of Epidemiology, School of Medicine in Katowice, Medical University of Silesia in Katowice, Katowice, Poland; 4 Department of Trauma and Orthopedics, District Hospital of Orthopedics and Trauma Surgery, Piekary Śląskie, Poland; 5 Department of Orthopedics and Traumatology, Sport-Clinic, Żory, Poland; 6 Metabolic Bone Diseases Unit, Department and Clinic of Internal Diseases, Diabetology and Nephrology, School of Medicine with the Division of Dentistry in Zabrze, Medical University of Silesia in Katowice, Zabrze, Poland

**Keywords:** Crude and standardized ratio, Age, Hip fracture, Osteoporosis

## Abstract

**Background:**

Patients with hip fractures present a great challenge for surgeons due to multimorbidity, polypharmacy as well as difficulty in communicating. These could be attributed to a recent trend in the aging patient population (80 years and older) as compared to the past. The aim of this study is to analyze age structure and location in male and female patients’ population with hip fracture over 50.

**Materials and methods:**

Hospital records between 2005 and 2014 with ICD-10 codes S72,0, S72,1 and S72,2 were included in the analysis. All fractures occurred in citizen aged 50 years and over living in the district of Tarnowskie Góry and the city of Piekary Śląskie in Poland.

**Results:**

Within the study period, 1258 hip fractures were registered. The mean age of the patients was higher every year, starting from 77.27 ± 9.52 in 2005 to 80.80 ± 9.65 years in 2014 (*p* < 0.01). The average age also increased in both gender groups from 73.85 ± 8.30 to 77.89 ± 9.52 years in male and from 78.14 ± 9.66 to 81.98 ± 9.49 years in female, respectively. The median age value was changed from 78.00 to 83.00 years in the total population. We noted a significant increase in female with trochanteric fracture; however, the level of neck fracture was almost the same. In men, crude rates for both trochanteric and cervical fractures slightly increased.

**Conclusions:**

As the age of patients increases, fractures were shown to be more complicated. Given the scale of the phenomenon and its determinants, we emphatically conclude orthogeriatrics is needed in Poland.

## Introduction

Hip fracture is one of the most common osteoporotic fractures. Nowadays, we can say that it is a worldwide challenge [1]. Orthopedic surgeons who work at Orthopedic and Traumatology Departments operate daily on patients with hip fracture (both cervical and trochanteric). Patients with fragility hip fracture are usually a great challenge for surgeons because of multimorbidity and polypharmacy, as well as difficulty in communicating. Due to the aging population, there are more patients aged 80 and older than in the past. These patients need a multidisciplinary approach, starting with geriatric and orthopedic care [2]. This type of system is called orthogeriatrics. According to a prospective, randomized, controlled trial, patients treated at specials orthogeriatric departments have better outcomes compared with the usual orthopedic [3]. Although the aging of polish population is well known there were no studies that show the trend of age of patients with hip fractures in Poland [4].

The aim of the study was to analyze age structure and location of hip fracture in male and female population, aged 50 years and over living in the district of Tarnowskie Góry and the city of Piekary Śląskie in a period of last 10 years from 2005 to 2014.

## Methods

The study covered the southern part of Poland, the district of Tarnowskie Góry and the city of Piekary Śląskie in Upper Silesia region. In 2014 (the last year of used for our observation), studied area was inhabited by 74947 residents aged 50 years and older (total population was 195,257 inhabitants, ~100 % of Caucasian population) [4]. Studied region composed of urban and rural dwellers in the proportion similar to the one of the total population of Poland. All patients (men and women) with musculoskeletal trauma are transported from the study area to Dr J. Daab Regional Hospital of Trauma Surgery in Piekary Śląskie. This is the only orthopedic hospital for the whole studied region so we may assume that almost all hip fractures sustained in the study population were managed in this institution. Case records of patients aged 50 years or older between the dates from January 1, 2005, and December 31, 2014, with code of the International Classification of Diseases ICD–10: S72,0 (cervical); S72,1 and S72,2 (intertrochanteric, subtrochanteric, inter and subtrochanteric fracture) were analyzed [5]. To assess the circumstances of the fall, we are based on anamnesis and the code ICD-10 (V01-Y98) of external causes of morbidity and mortality, and only fragility fractures (caused by the falls from a standing height or less) were included to analysis [5]. Other cases like: patients with address outside of studied area, and history of non low-energy fracture (e.g., car accident, falls from more than standing height) were excluded from the study. Each fracture was confirmed by an X-ray, and in justified cases a CT scan was performed. Cases of transfers to another hospital and readmission were identified. Records were analyzed to exclude duplicate records from the final data set. In order to confirm the goal of the study, we first calculated the crude rates for hip fracture per 100,000 population for the district of Tarnowskie Góry and city of Piekary Śląskie. Similarly, crude-specific rates were calculated for studied events in male and female population. Patients were divided into age groups as follows: 50–59; 60–69; 70–79; 80+ years. Our initial observation began in 2005 which also happens to be the year the Central Statistical Office of Poland published, for the first time official data for the oldest group (80+ years) [4].

### Statistic

Demographical data including number of people by gender, age and place of residence were obtained from the official database available from the website of the Central Statistical Office in Warsaw [4]. First, we described subjects using simple descriptive statistics typical for qualitative and quantitative variables. Differences between age of patients in particular gender group, type of fracture or study year were assessed using Mann–Whitney U test. Next, we calculated the crude rates of total hip fracture and separate neck or trochanteric fracture in total population in each year of the study period. We then used the directly standardized procedure to calculate standardized rates for each type of fracture in particular years based on the principles adopted in epidemiology. We used the Segi-world population as the standard population [6]. Moreover, the 95 % confidence intervals (CIs) were calculated for each aged groups in particular years, assuming Poisson distribution and gamma distribution when the number of incidences was small. Interpretation of statistical significance was based on *α* = 0.05 criterion. All analyses were performed using SAS 9.4 (SAS Institute Inc., Gary, NC).

## Results

In study period, there were 1258 patients with fragility hip fracture aged from 50 to 101 years. Mean age of subjects was 78.59 ± 10.34 years, while the median age was 80 years. Most of the study groups were women (*n* = 921; 73.21 % of subjects).

### Age

**Fig. 1:**
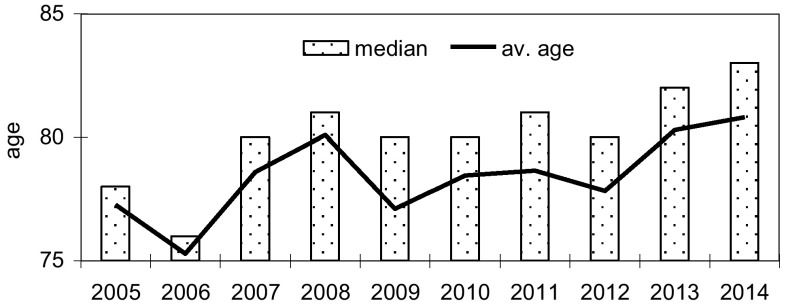
Average age of patients and median in years 2005–2014

Mean age of patients in the first year (2005) was statistically significant lower (*p* < 0.01) than those in the last year (2014) of the study, 77.27 ± 9.52 and 80.80 ± 9.65 years, respectively. Average age increased in both gender groups from 73.85 ± 8.30 to 77.89 ± 9.52 years in male (*p* = 0.06) and from 78.14 ± 9.66 to 81.98 ± 9.49 years in female (*p* < 0.01), respectively. The median value was changed from 78.00 to 83.00 years in total population and in male from 74.00 to 80.00 years, and in female from 79.00 to 84.00 years. This is shown in Fig. 1.

**Fig. 2:**
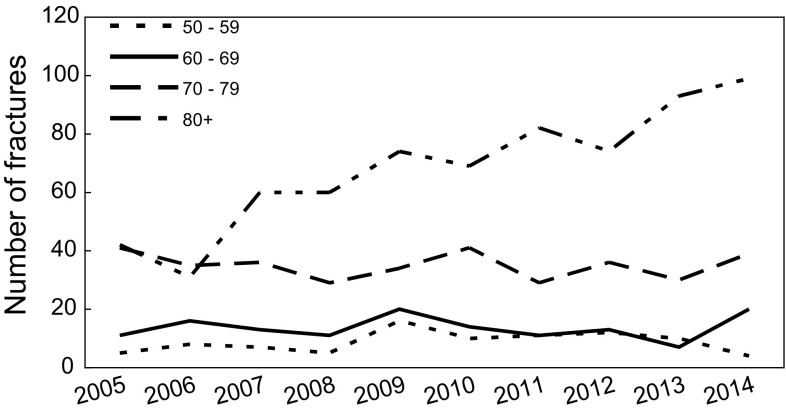
Number of fractures in aged groups in particular years of the study period

**Fig. 3:**
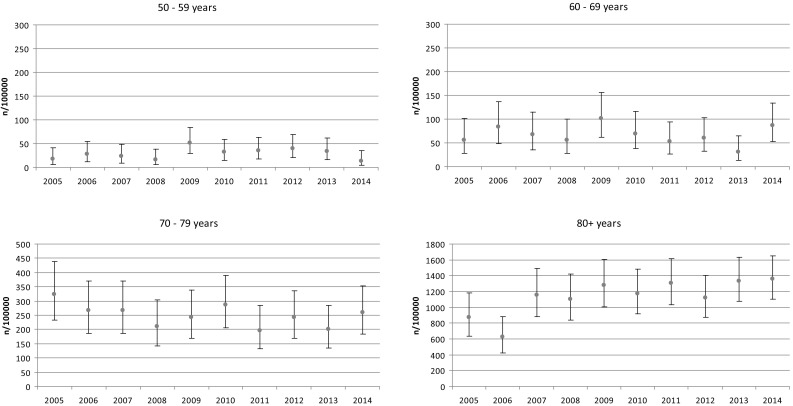
Changes in hip fracture incidence per 100,000 population (crude value and its 95 % CI) in particular aged groups during the study period (2005–2014)

When age is the factor that is used, the data showed most fractures (54 %) to occur in group of patients 80 years and older. During the 10 years of our study, we observed the tendency of increase the total number of fracture in this group only (Fig. 2) as well as crude rates (Fig. 3).

### Location

**Fig. 4:**
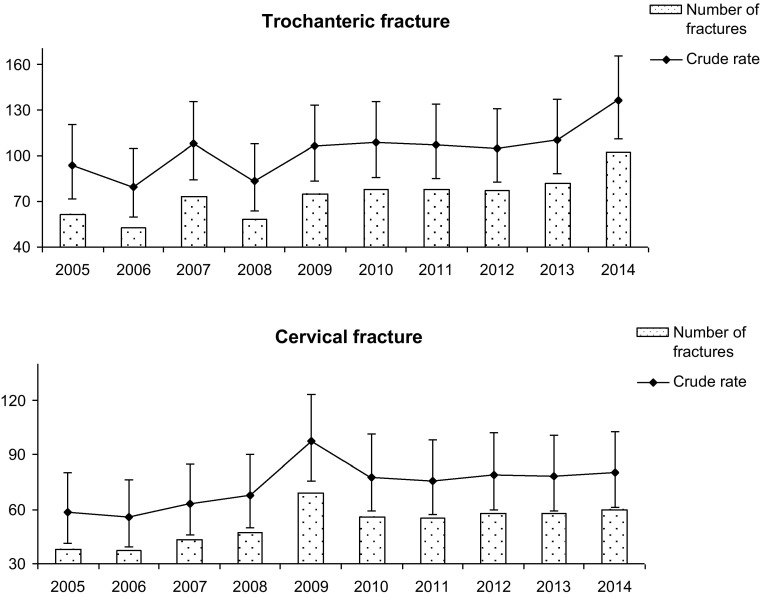
Number of cases and crude rates for hip fracture with their 95 % CI in particulars years of the study period for cervical and trochanteric fractures

**Fig. 5:**
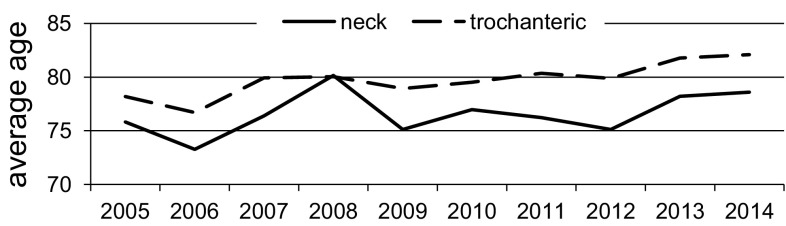
Average age of patients with trochanteric and cervical fracture in years 2005–2014

In the studied group, there were 521 (41.41 %) femoral neck fractures and 737 (58.59 %) trochanteric fracture. During our observation, the number of trochanteric fractures increased 2.4 fold, while femoral neck increases by 1.7 fold (Fig. 4). Table 1 and Fig. 4 show the values of number of cases, crude and standardized rates for hip fracture with their 95 % CI in study years period for cervical and trochanteric fractures. Older patients have greater odds ratio for trochanteric fracture (Table 2). The patients with femoral neck fractures were younger (76.65 vs. 79.96 year, *p* < 0.01). When gender is considered, the average age of men with fracture was 73.70 ± 11.28 years (73.51 and 73.90 years for trochanteric and femoral neck fracture, respectively; *p* > 0.05). The average age in women was higher 80.38 ± 9.37 years (82.00 and 77.85 years, respectively; *p* < 0.01). The differences between genders were statistically significant (*p* < 0.01). The average age of patients with each type of fracture slightly increased in the study period. The tendency is almost parallel (Fig. 5).

**Table 1:** Standardized rates for cervical and trochanteric fractures per 100,000 population with their 95 % CI in particulars years of the study period

Year	2005	2006	2007	2008	2009	2010	2011	2012	2013	2014
Cervical fracture	47.1 (33.1–65.1)	49.7 (34.6–69.2)	50.2 (36.0–68.1)	50.9 (37.1–68.3)	78.6 (60.4–100.5)	61.1 (45.6–80.2)	57.9 (42.9–76.3)	60.2 (45.1–78.7)	53.7 (40.2–70.4)	55.5 (41.7–72.5)
Trochanteric fracture	73.2 (55.7–94.4)	63.1 (46.9–83.1)	82.0 (63.8–103.7)	62.9 (47.3–82.1)	80.2 (62.5–101.4)	79.1 (62.1–99.4)	71.3 (55.9–89.5)	70.8 (55.3–89.3)	69.0 (54.4–86.3)	87.5 (70.5–107.2)

**Table 2:** Odds ratio of each type of fracture in specific age groups

Type of fracture	Age group	Odds ratio	CI 95 %	*p*
Cervical	50–59	1.65	1.18–2.32	<0.01
	60–69	1.23	0.93–1.63	0.15
	70–79	0.79	0.64–0.98	0.03
	80+	0.62	0.52–0.75	<0.01
Trochanteric	50–59	0.61	0.43–0.85	<0.01
	60–69	0.81	0.61–1.08	0.15
	70–79	1.27	1.02–1.57	0.03
	80+	1.60	1.33–1.93	<0.01

**Fig. 6:**
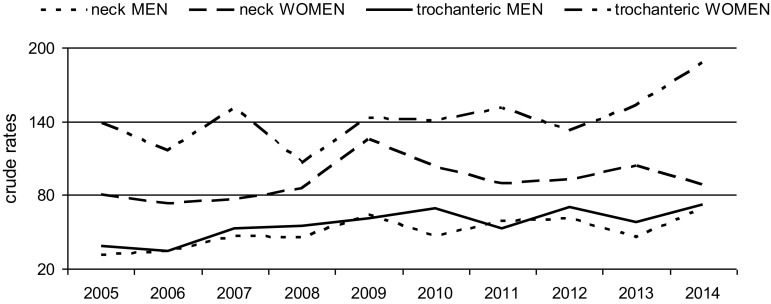
Crude rates of trochanteric and cervical fractures (n/100,000) in men and women in study period

During our observation, a significant increase in female trochanteric fracture was noted. However, the level of rest neck fracture was almost unchanged. In men, crude rates for both trochanteric and cervical fractures slightly increased (Fig. 6).

## Discussion

### Aging population

The aging population presents a worldwide challenge. From 1950 to 2000, the percentage of people aged 65+ increased from 8 to 12.6 % in the USA alone [7]. Likewise in the European Union, between the years 2004–2014 the population had increased from 16.4 to 18.5 % [8]. A similar situation is also observed in Poland. The proportion of elderly population in 2000 was 12.4 %, in 2010 13.5 %, while it is estimated to be approximately 27 % in 2030 [9].

Unfortunately, to make matters worse together with the growth of number of people 65+ years, decline of the total number of population is projected [4]. Life expectancy by age in Poland from 1950 to 2014 in group 60+ was changed from a value of 14.6–19.2 years for men and from 19.3 to 24.3 years for women [4]. Consequently, the oldest group (age more than 65 years) had increased from 5.076 million in 2005 to 5.874 million of population in 2014 [4]. However, younger group (15–64 years) had decreased from 26.892 million to 26.840 million [4]. A similar trend was observed in our studied region. From 2005 to 2014, the number of citizen 50+ years had increased from 65016 to 74947. Meanwhile, the total number of residents was reduced by 2646 persons [4].

The increase in life expectancy causes more and more elderly population to sustain fracture. Also, the mean value of aged patients with a diagnoses of fracture increases. The median age of our patients increased from 78 to 83 years in study period. In comparison with our previous study, the mean value of age is also higher (from 77.57 to 78.59 years) [6].

In our study, the oldest age group was the most numerous. This tendency is similar in other countries [10–12]. As evident in group under 80 years, there is no increase in the number of fracture. This may be due to the fact that people under the age of 80 are mostly strong enough to avoid falls and their bones are still in good quality [13–15]. The problem begins in the oldest group (80+ years). In our study, we observe a high increase in fracture only in this age group. People above 80 years are not strong enough to lead an independent life. Often, they prefer to do many things themselves but may lack the muscle strength to perform such tasks due to factors such as diseases that precipitates falls and in consequence fractures [15, 16]. Furthermore, older people actually often live alone without rest of their younger family. It imposes that they perform most of the things themselves making them more likely to falls. In our country, older people are mostly poor and cannot afford a housekeeper [7]. In this group, there are more frail persons that have an increased risk of recurrent falls and fractures [17]. The dominant group among subjects is patients with fracture in the past, which is the major prognostic factor for the next one [18].

### Location of fracture

In our study, we observed a higher increase in trochanteric fracture. It is a worldwide challenge [19, 20], and this could probably be attributed to an every aging population. Our observation is similar to published data. On average, patients with trochanteric fractures are older than those with cervical ones [20–23]. This tendency was not observed in male population. The mean value of age was similar to those noted in previous studies [24, 25], probably because men have a shorter lifespan than women [4]. Seeley in his prospective study observed that low-appendicular bone mineral density was a strong risk factor for intertrochanteric but not femoral neck fractures [26]. Also Vega reported that females with trochanteric fractures are older, thinner and have lower BMD (*bone mineral density*) in all measured sites [27]. The lower BMD in patients with trochanteric fracture was also confirmed by other researchers [28–30] as well as the lower quantitative ultrasound parameters [31]. According to a study of Uitewaal trabecular bone volume and trabecular surface density, there were significantly lower in trochanteric fractures than in cervical [32]. Also, mean trabecular plate thickness and mean wall thickness were significantly lower in trochanteric fractures. He concluded his analysis by stating that *trochanteric fractures are associated with serious osteoporosis, whereas cervical fractures constitute a more heterogeneous group* [32]. Also in EPIDOS study, low total body BMD or QUS (*quantitative ultrasounds*) parameters were not significant predictors of cervical fractures [31].

In a later study based on quantitative computed tomography (QCT), no significant differences were found in trabecular BMD between types of fracture in the studied region (middle of the intertrochanteric region, the point of minimum femoral neck cross-sectional area and center of the femoral head). The limitation of these studies cited above was the fact that females and males were included together in one group. However, patients with trochanteric fractures had a smaller cortical index at the level of femoral canal isthmus and smaller neck shaft angle. Researchers also confirmed that trochanteric fracture is related to severe osteoporosis (with thinner cortical bone of the femoral diaphysis) [33].

In cervical fracture, except BMD and QUS parameters, other risk determinants are considered such as high BMI (*body mass index*), antihypertensive therapy, a large body height and fat percentage, steroid use, impaired functional status and pelvic structure [21, 34–36].

The increase in fracture was observed especially in women in “trochanteric” group. The tendency in men was slightly higher but constant. It is caused by a stronger age-related decrease in BMD in women [37].

Generally, in Poland, we observed an increase in the number of old patients with severe osteoporosis leading to more trochanteric fractures. Unfortunately, this type of fracture is more challenging, especially when is comminuted and unstable type of fracture. Furthermore, it is an absolute indication for surgery in comparison with femoral neck fracture [38].

Given the scale of the phenomenon and its determinants, we emphatically concluded that we need orthogeriatrics in Poland. Nowadays, in our health system, there is no special system of health care for elderly patients. There are only 12 departments of geriatrics in Silesian voivodeship (902 781 citizens above 65 years) [4, 39]. As the age of patients increases, so are the many disease states in elderly and the complicated natures of the fractures (i.e., more trochanteric fracture). Older patients are weaker and require the need for more attention and rehabilitation. These patients need a department with geriatricians, orthopedic surgeons, physiotherapists and psychologists. They may need to be transferred to a special rehabilitation department immediately after surgery. Each patient should have an access to their own osteoporosis-coordinator who will try to explain problems associated with osteoporosis, further treatment (how and where treat osteoporosis), prevention (participation in special programs etc.) and to help patients and their families prevent further falls.
